# Ultrafast and Sensitive Screening of Pathogens by Functionalized Janus Microbeads‐Enabled Rotational Diffusometry in Combination with Isothermal Amplification

**DOI:** 10.1002/smsc.202200010

**Published:** 2022-03-03

**Authors:** Dhrubajyoti Das, Hui-Chen Hsieh, Chang-Shi Chen, Wei-Long Chen, Han-Sheng Chuang

**Affiliations:** ^1^ Department of Biomedical Engineering National Cheng Kung University Tainan city 70101 Taiwan; ^2^ Department of Biochemistry and Molecular Biology National Cheng Kung University Tainan city 70101 Taiwan; ^3^ Institute of Basic Medical Sciences College of Medicine National Cheng Kung University Tainan city 70101 Taiwan; ^4^ Medical Device Innovation Center National Cheng Kung University Tainan city 70101 Taiwan

**Keywords:** DNA, *Escherichia coli*, functionalized Janus microbeads, loop-mediated isothermal amplification, nucleic acid, pathogen screening, rotational diffusometry

## Abstract

Polymerase chain reaction (PCR) is widely considered the gold standard in molecular diagnostics because of its high accuracy. However, conventional PCR is very time‐consuming (turnaround time 4–6 h), labor‐intensive, and high‐tech instruments dependent. Therefore, developing an ultrafast and sensitive nucleic amplification‐based detection method is still in high demand. Herein, a rapid screening of *Escherichia coli* using an advanced and sensitive biosensing method known as rotational diffusometry in combination with loop‐mediated isothermal amplification (LAMP) is reported. Given that the solution viscosity is increased with nucleic acid amplification, the viscosity change can be measured by the functionalized Janus microbeads‐enabled rotational diffusometry. The rotational diffusivity derived from capturing images of microbeads is expressed in terms of blinking signals. Under such conditions, this method can achieve a limit of detection of 42.8 fg μL^−1^ in 10 min for *E. coli* with sample volume as low as 2 μL. Additionally, detection of *E. coli* whole cells extracted from artificially contaminated milk, juice, and water is performed by the developed method and validated with real‐time PCR. Through this biosensing technique, a rapid, reliable, and low sample volume screening tool can be established, improving the shortcomings of the current time‐consuming and labor‐intensive methods.

## Graphical abstract

Herein, rotational diffusometry is combined with loop‐mediated isothermal amplification (LAMP) in a microfluidic assay with functionalized Janus particles. In presence of LAMP‐amplified DNA, the solution viscosity increases. This leads to a lower rotational diffusion with a generation of a slow‐blinking signal of the particles. However, without amplified DNA, the sample viscosity remains constant. Hence, the particles exhibit a fast‐blinking signal.

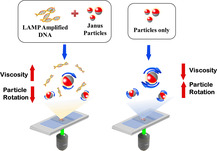

## 1 Introduction

Rapid and sensitive screening of pathogens is an important tool in the early management of infectious diseases. Extremely low concentrations of pathogens in an early stage of disease presents unique challenges in rapid diagnosis.^[^ ^1^ ^]^ Nucleic acid amplification tests (NAATs) play an important role in providing highly selective and ultrasensitive detection of pathogens, via enzymatic amplification of some specific nucleic acid sequence, enabling the detection of trace amounts of viral or bacterial NA in the very early stage.^[^ ^2^, ^3^ ^]^ In molecular diagnostics, polymerase chain reaction (PCR) is considered the current gold standard due to its high specificity and sensitivity. However, conventional PCR requires a high‐tech laboratory set up with expensive instrumentation, complex thermal cycling process, and trained personnel, which impedes its role in rapid screening.^[^ ^4^ ^]^ Hence, developing an ultrafast nucleic acid amplification technique becomes an urgent need to win the battle.

In the past decades, several next‐generation biosensors based on colorimetry, fluorimetry, surface plasmon resonance, and electrochemistry have been developed for the rapid detection of pathogens with high sensitivity and selectivity.^[^ ^5^, ^6^ ^]^ For example, functionalized quantum dots and other fluorescent signal transducers have been used substantially to detect *Escherichia coli* in the range of 450–10^7^ CFU mL^−1^, *Mycobacterium tuberculosis* at 10^4 ^CFU mL^−1^, and *Staphylococcus aureus* at 9.4 × 10^4^ CFU mL^−1^.^[^ ^7^, ^8^, ^9^, ^10^, ^11^, ^12^ ^]^ Additionally, nanoparticles‐based colorimetric biosensors have been shown to have a limit of detection of 10^2 ^cells mL^−1^ for *E. coli*, 4.4 × 10^1^ CFU mL^−1^ of *Salmonella pullorum,* and 4.4 × 10^1^ cells mL^−1^ of *Listeria monocytogenes*.^[^ ^13^, ^14^, ^15^ ^]^ Further, optical and spectroscopic‐based biosensing techniques such as surface‐enhanced Raman spectroscopy (SERS), and dark‐field microscopy techniques also show potential in pathogen detection application.^[^ ^16^, ^17^ ^]^ Zhou et al. proposed a method that involved coating of the silver nanoparticles on the bacterial cell wall for *E. coli* detection with the limit of detection (LoD) of 2.5 × 10^2^ cells mL^−1^ based on SERS.^[^ ^18^ ^]^ Xu et al. reported *E. coli* detection in 30 min using antibodies conjugated gold nanoparticles by an entry‐level dark‐field microscope.^[^ ^19^ ^]^ Though these technologies are promising, still they struggle to overcome all of the challenges incurred in pathogen detection as these methods require large‐scale pre‐processing, high‐tech instruments, and expensive labels for detection, impeding its application in resource‐limited regions. Indeed, designing integrated biosensors that provide accurate and fast detection of infectious pathogens with high sensitivity is still in demand.

**Figure 1:**
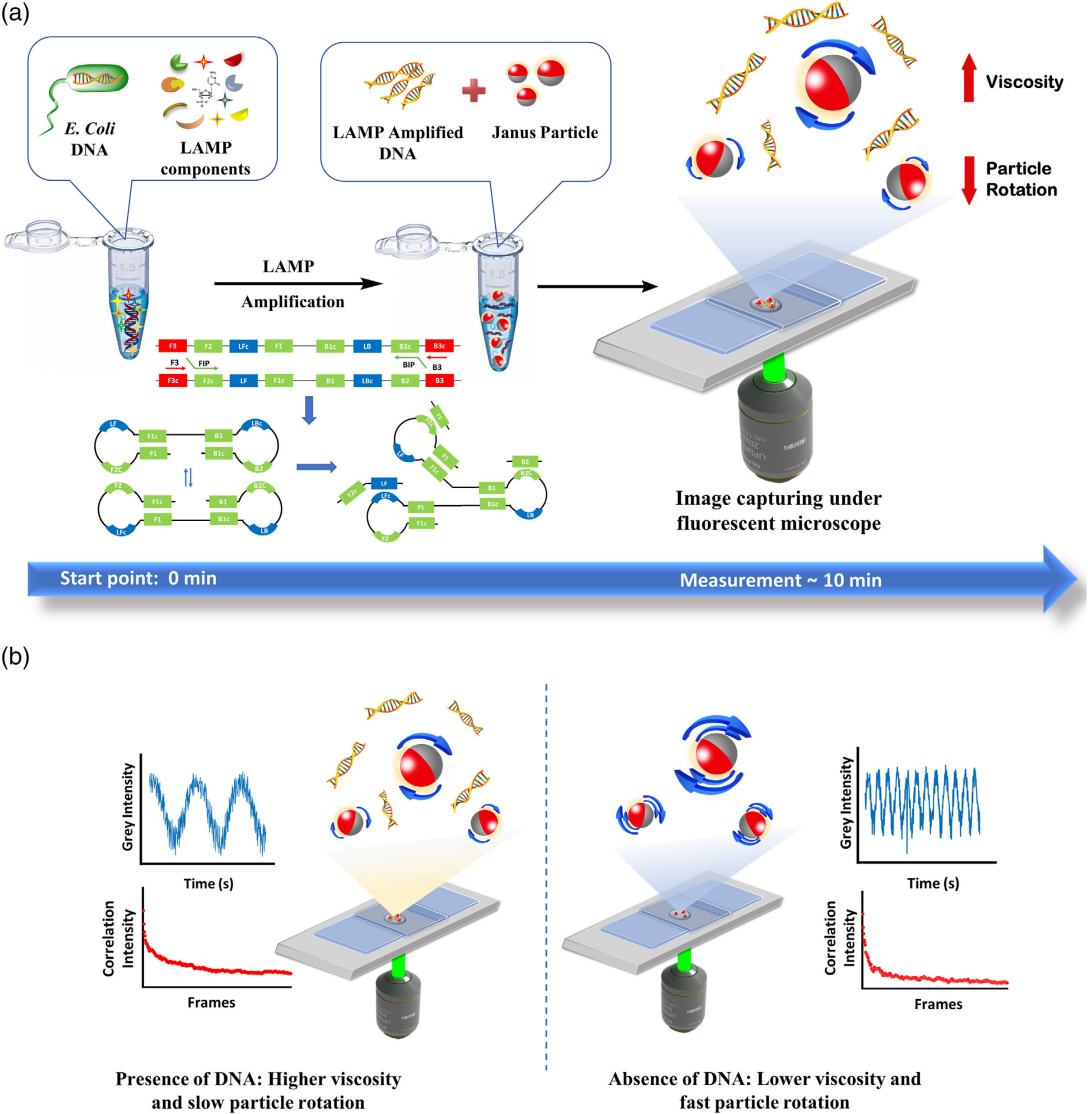
a) Schematic illustration of *E. coli* detection method enabled by rotational diffusometry combined with LAMP amplification using functionalized Janus particles. LAMP amplification of *E. coli* DNA was performed by mixing the appropriate concentration of selected primers, LAMP components, and *E. coli* genomic DNA. 2 μL of the suspension of LAMP‐amplified sample and Janus particles and was pipetted on the glass slide. The Janus particles were observed under an IX71 fluorescent microscope with a 10× objective lens followed by the image capturing with a charge‐coupled device (CCD) camera and data analysis. b) Conceptual illustration of the working mechanism of rotational diffusometry in microfluidic assay with modified Janus particles. In presence of LAMP‐amplified DNA, the solution viscosity increases, which leads to a lower rotational Brownian motion of the particles and slow blinking signals while in absence of LAMP‐amplified DNA, the Janus particles show higher rotational Brownian motion with fast blinking signal due to the lower viscosity of the solution.

Loop‐mediated isothermal amplification (LAMP) is an alternative and attractive technique for rapid and cost‐effective nucleic acid amplification with high accuracy.^[^ ^20^, ^21^, ^22^, ^23^ ^]^ Detection of amplified products has been integrated into LAMP assays using electrochemical, fluorescent, and visual methods.^[^ ^24^, ^25^, ^26^ ^]^ Due to its ease of use, robustness, and ability to mitigate the use of complex thermal cycling processes, LAMP is currently being implemented in rapid diagnostics.^[^ ^27^ ^]^ Combining the promising advantages of LAMP and our previous achievements on diffusometric method,^[^ ^28^, ^29^, ^30^, ^31^, ^32^, ^33^ ^]^ we wish to develop a novel biosensor to carry out a rapid, simple, and quantitative detection of pathogens using Janus particles to quantify the rotational Brownian motion, using *E. coli* as a proof of concept (**Figure** **1**a). According to the Stokes–Einstein–Debye relation, the rotational diffusivity is inversely proportional to the liquid viscosity.^[^ ^34^, ^35^ ^]^ LAMP polymerization produces up to ≈10^9^ copies of DNA targets in less than an hour with various lengths up to 25 kilobases.^[^ ^21^ ^]^ These large numbers of amplified nucleic acid increases the solution viscosity followed by a significant decrease in Brownian motion of microscopic particles.^[^ ^36^, ^37^, ^38^ ^]^ The decrease in diffusivity, yields a slow blinking signal of the functionalized Janus particles.^[^ ^39^, ^40^, ^41^, ^42^ ^]^ However, in absence of bacterial genome, LAMP amplification is inactive, hence the sample viscosity remains stable. Thus, the diffusivity coefficient as well as the blinking signals generated by the particles stay constant (Figure 1b). This technique relies on a fairly intuitive method without additional power and failure problems to identify the presence of pathogenic nucleic acids. To the best of our knowledge, this is the first to attempt a rapid and sensitive detection of pathogens using rotational diffusometry combined with the LAMP technology. Because of the ultrasensitive rotational Brownian motion, we achieved the LoD as low as 42.8 fg μL^−1^ in 10 min with a sample volume of 2 μL. Upon successful development of this ultrafast screening technique may raise an important impact on efficient detection of on containing highly contagious diseases like COVID‐19 in a timely fashion.

## 2 Results and Discussion

### 2.1 LAMP Amplification of *E. coli*

**Figure 2:**
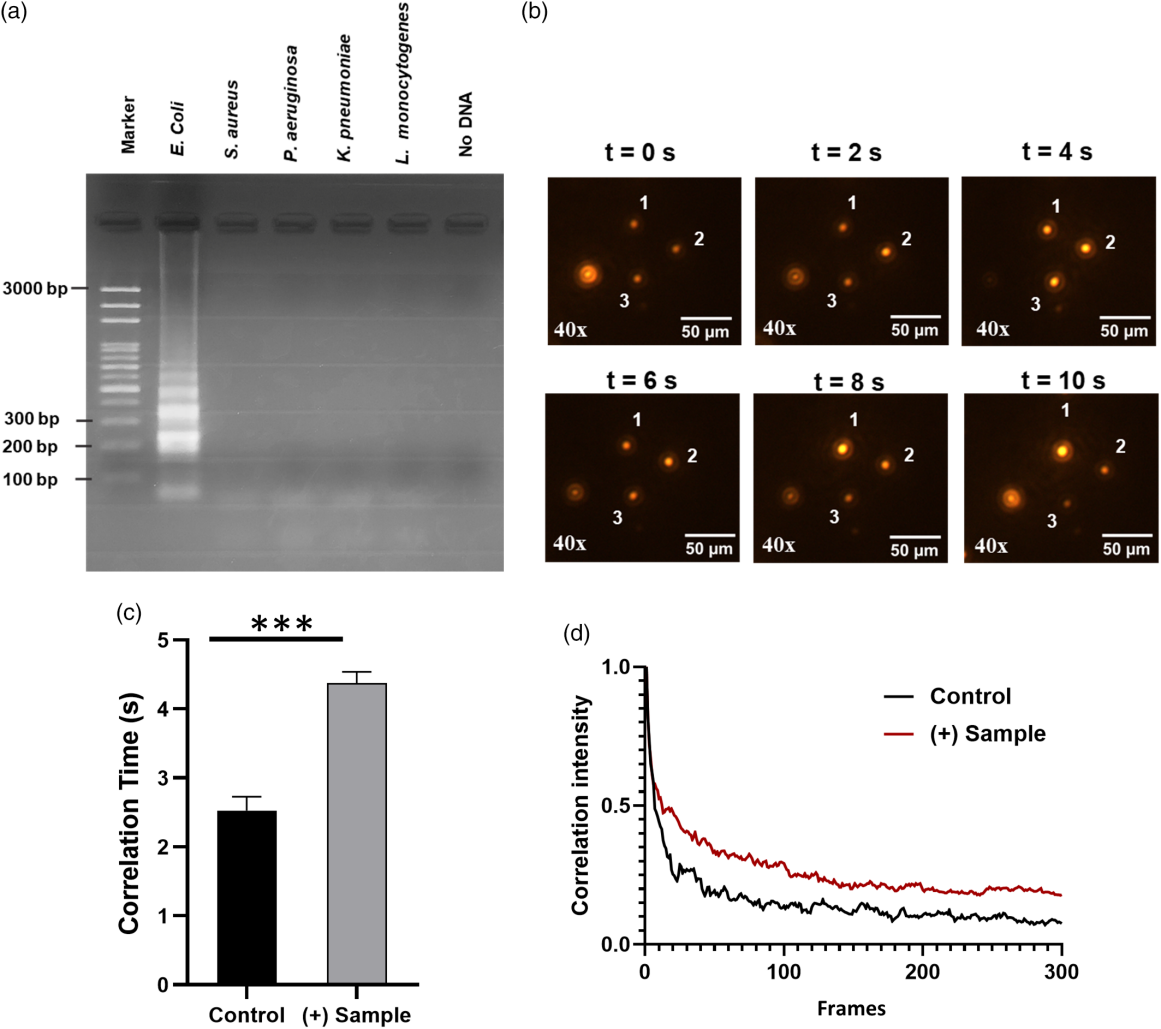
Detection of *E. coli* DNA amplification and specificity test. a) Specificity test of the LAMP primers and their comparison with various control strains. No amplification was observed for the control strains. b) Image sequence of 1 μm Janus particles under a fluorescent microscope (40×). The numbers mark the tracked particles in different image frames. c) Correlation time plot of LAMP‐amplified DNA of *E. coli* compared with the control. Here the LAMP reaction is performed at 70 °C where the (+) sample is undergoing LAMP reaction with *E. coli* genomic DNA and the control underwent LAMP reaction without any genomic DNA. d) Exponential decay of cross‐correlation intensity. The black and red curves refer to the control and (+) sample, respectively. (***p < 0.001, *n* ≥ 3).

*E. coli* was used as a model microorganism to evaluate the performance of this method. The *uidA* gene of *E. coli* was analyzed in this study and the specificity of the LAMP primers were tested with *Klebsiella pneumoniae,* *S. aureus, Pseudomonas aeruginosa,* and *L. monocytogenes.* The DNA templates were extracted from each bacterium and then adjusted to the same concentration. The amplified target DNA fragments of the bacteria were initially identified by 1.5% of agarose gel electrophoresis. No other peaks were observed in the LAMP products apart from the *E. coli* confirming the selectivity of the primers (**Figure** **2**a). The optimum amplification was observed at 70 °C (Figure S1, Supporting Information), therefore, all the LAMP reactions in this work were performed at 70 °C.

To validate that rotational diffusometry could be used to identify LAMP amplification, a series of experiments were performed incubating 1 μm of modified Janus particles in the LAMP‐amplified DNA solution. Blinking images of Janus microbeads (Figure 2b) were recorded for each sample by placing six droplets on a microchip setup with a distance of 200 μm apart followed by analysis using a cross‐correlation algorithm. During all the experiments, the microchip temperature was maintained at 25 °C. The evaluation of the rotational diffusometry method and synthesis and characterization of Janus particles were well explained in our previous work.^[^ ^39^, ^40^ ^]^ The sample containing amplified *E. coli* genomic DNA (10 ng μL^−1^) was correctly identified with a statistically significant difference in the values (*p* < 0.001). Data from this study presented in Figure 2c shows that the sample containing LAMP‐amplified DNA (+ Sample) had a relatively higher correlation time as compared to the control (underwent LAMP reaction without any genomic DNA), indicating the LAMP‐amplified product increased the fluid viscosity. Additionally, the correlation intensity versus image frames plot was displayed as an intensity diagram in Figure 2d for the control and the sample containing LAMP‐amplified DNA, where an exponential decay in the correlation intensity was observed with the elapsed time because of loss of pairs. (+) Sample containing LAMP‐amplified DNA showed a slow decline in the correlation intensity due to weak diffusion, whereas, in the case of control, a strong diffusion rapidly brought down the correlation intensity.

### 2.2 Effect of LAMP Reaction Time on Rotational Diffusometric Measurements

**Figure 3:**
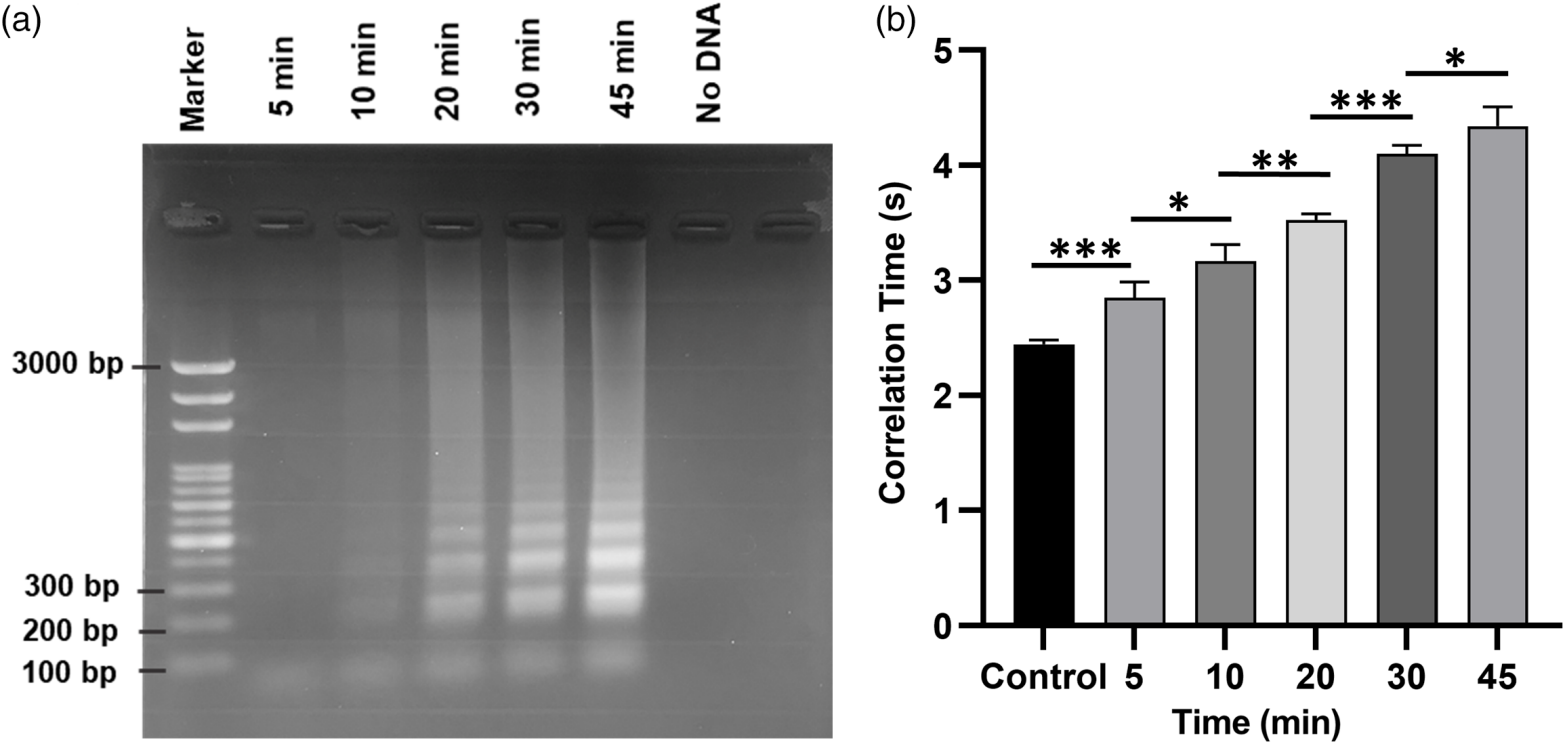
Detection of *E. coli* with respect to various LAMP reaction times. a) Gel electrophoresis image of LAMP‐amplified *E. coli* DNA with the change in reaction time. b) Correlation time plot of LAMP‐amplified *E. coli* DNA with respect to different reaction times. (*p < 0.05, **p < 0.01, ***p < 0.001, *n* ≥ 3).

To understand the effect of time on DNA amplification, a series of LAMP reactions were performed with different times ranging from 5 to 45 min with *E. coli* genomic DNA concentration of 10 ng μL^−1^. Gel electrophoresis was used as well to validate the DNA amplification, where the images of amplified samples show an increasing trend in the band intensity with the increase of reaction time (**Figure** **3**a). This indicates the increase in LAMP‐amplified product with the increase in reaction time. As expected, a higher correlation time was observed for the longer reaction time, showing a gradual increasing trend in the correlation time as well. Figure 3b shows a relative statistically significant difference (*p* < 0.001, *n* ≥ 3) in the correlation time between the particles suspended in the LAMP‐amplified DNA sample in comparison with the control, indicating the successful detection of *E. coli* within 5 min. This proves that the rotational diffusometry is an ultrafast method for the detection of nucleic acid amplification.

### 2.3 Evaluation of Detection Sensitivity

**Figure 4:**
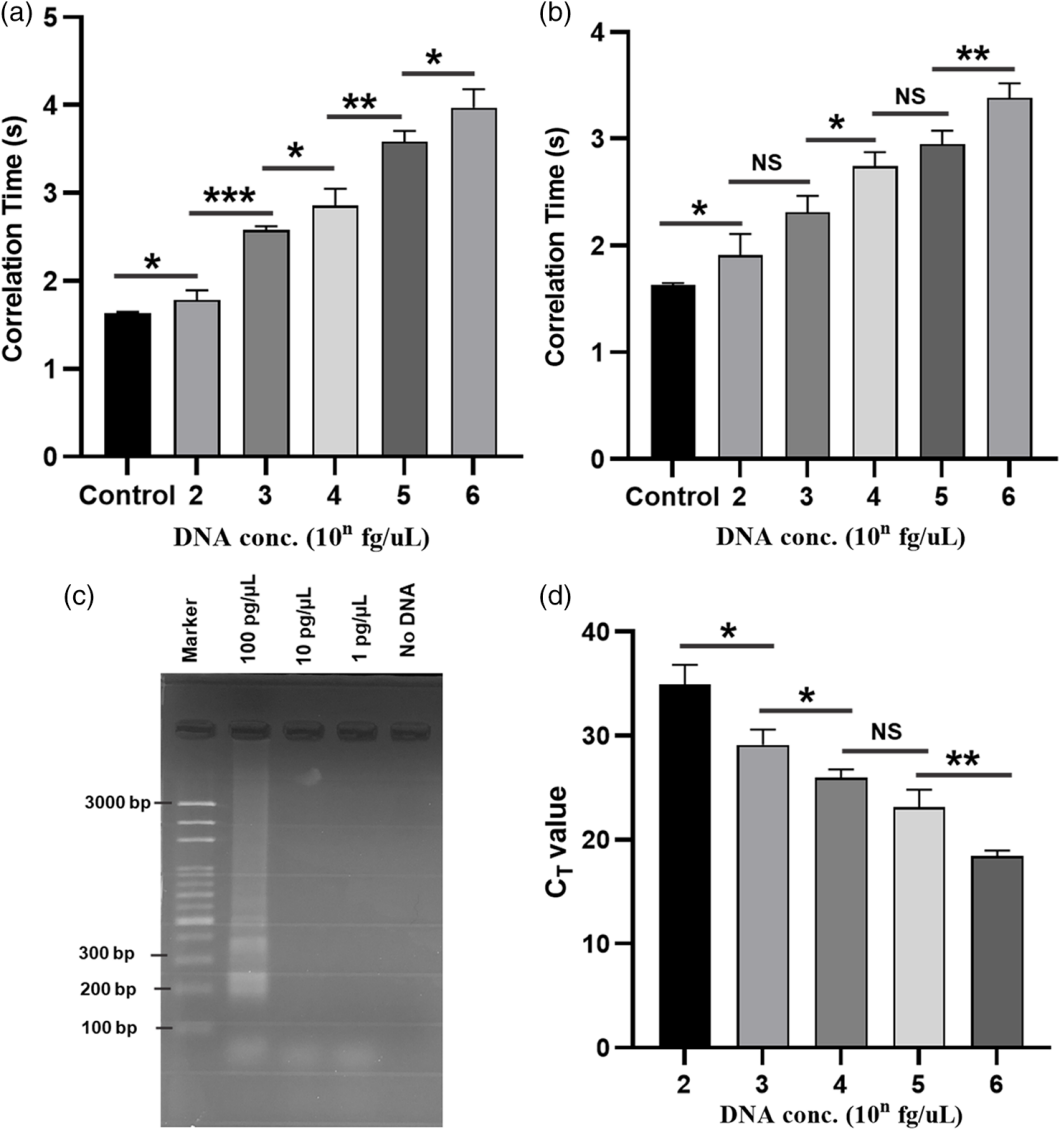
Correlation time plot of LAMP amplification of *E. coli* with respect to different DNA concentrations ranging from 100 fg μL^−1^ to 1 ng μL^−1^ for different reaction time a) LAMP amplification for 10 min. b) LAMP amplification for 20 min. c) Gel electrophoresis image of 20 min LAMP amplification in different of *E. coli* genomic DNA concentrations. d) C_T_ value plot of real time PCR amplification of *E. coli* with different DNA concentration ranging from 100 fg μL^−1^–1 ng μL^−1^. (*p < 0.05, **p < 0.01, ***p < 0.001, ****p < 0.0001, *n* ≥ 3).

LAMP polymerization was performed with different *E. coli* genomic DNA concentrations ranging from 100 fgμL^−1^ to 1 ng μL^−1^ with 20 and 10 min reaction period. The LAMP‐amplified products were identified using rotational diffusometry. Samples with higher DNA concentration showed significantly higher amplification resulting in larger viscosity change. A statistically significant higher correlation time (*p* < 0.01, *n* ≥ 3) was observed compared to control for 10 min LAMP reaction (**Figure** **4**a). The LoD was eventually estimated to be 42.8 fg μL^−1^, which was defined as the intersection of the trend line of the concentration and the three standard deviations of the control. A statistically significant higher correlation time was observed compared to control (*p* < 0.05, *n* ≥ 3) for the samples after a 20 min LAMP reaction (Figure 4b). DNA amplification was also confirmed by gel electrophoresis, where the detection sensitivity was achieved 100 pg μL^−1^ for a 20 min LAMP reaction (Figure 4c). We have validated our findings with real‐time PCR (Figure 4d) and the results appear to be in good agreement with the rotational diffusometric assay. The C_T_ value of 34.9 was obtained in real‐time PCR analysis for 100 fg μL^−1^ *E. coli* DNA concentration, which makes the total analysis time of 1 h 50 min. This indicates that the analysis time is improved 11‐fold by our method in comparison with real‐time PCR, without compromising the detection sensitivity. After analyzing these results, it was noteworthy to mention that rotational diffusometry with LAMP is an ultrafast method for the detection of pathogens with high sensitivity.

### 2.4 A Preliminary Test with Water and Food Samples

**Figure 5:**
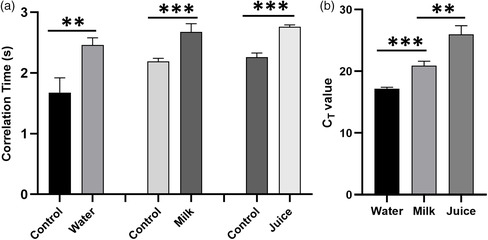
a) Rotational diffusometry measurements of correlation time change of 10 min LAMP amplification of *E. coli* whole cells extracted from artificially inoculated water, milk, and juice. b) C_T_ value plot for real‐time PCR analysis of *E. coli* whole cells in artificially inoculated water, milk, and juice (**p < 0.01, ***p < 0.001, *n* ≥ 3).

*E. coli* is usually found in water and food items like milk and fresh juice, thus it is essential to perform the *E. coli* detection in different matrices. Molecular biology grade water and the food samples were spiked with *E. coli* and cultured for several hours. Extracted bacterial cells from each of the samples were redissolved in molecular biology grade water and used for LAMP reaction. 5 μL samples were directly used for the LAMP reaction. Gel electrophoresis was used to confirm the DNA amplification in water, milk, and juice (Figure S2, Supporting Information). **Figure** **5**a shows a relative statistically significant difference (*p* < 0.01, *p* < 0.001, *p* < 0.001, *n* ≥ 3) in the correlation time between the artificially inoculated samples with *E. coli* cells in comparison with the control (without spiking *E. coli* cells), indicating the successful detection of *E. coli* within 10 min in all the three matrices. We have validated our findings with real‐time PCR (Figure 5b) and the real‐time PCR results appear to be in good agreement with diffusometric data. Eventually, the preliminary test and the validation confirm the potential of rotational diffusometry for future use of routine food microbial analysis.

## 3 Conclusion

As of today, in molecular diagnostics, PCR remains the gold standard for many sample detections due to its high accuracy. However, conventional PCR is still limited by long time‐consumption, high labor‐intensiveness, and heavy high‐tech instrument dependence. Therefore, to carry out rapid, simple, and quantitative nucleic acid amplification, we proposed using Janus microparticles to measure the rotational Brownian motion combined with LAMP technology. In this work, we demonstrated that rotational diffusometry with the aid of LAMP can be used as a rapid and sensitive tool for pathogen detection where we use *E. coli* as a proof of concept. We achieved an LoD of 42.8 fg μL^−1^ in 10 min with a sample volume as low as 2 μL. Our assay facilitated sensitive analysis of *E. coli* whole cells extracted from artificially contaminated milk, juice, and water, while the same samples were validated with real‐time PCR. In conclusion, these results showed that the rotational diffusometry is a definitive method for ultrafast screening of pathogens. Eventually, we look forward to advancing this technology to provide a benchtop platform for robust, sensitive, and selective detection of contagious pathogens.

## 4 Experimental Section

4.1

4.1.1

##### Bacterial Culture Growth and Genomic DNA Extraction

*E. coli* (ATCC 25 922), *S. aureus* (ATCC 23 360), *L. monocytogenes* (ATCC 19 115), *K. pneumoniae* (ATCC 700 603), and *P. aeruginosa* (ATCC 27 853) were purchased from American Type Culture Collection (Manassas, VA, USA) and their frozen stocks (20% glycerol and 5 mL of TS broth) were used for the culture preparation. The bacterial culture was grown in TS broth (TSB; 211 825, BD, Sparks, MD, USA) at 37 °C for 24 h. Gene Spin genomic DNA extraction kit (PT‐GD112, Pro‐Tech chemicals, Taiwan) was used to extract and purify the genomic DNA from the prepared bacterial culture. Genomic DNA extraction for gram‐negative (*E. coli, P. aeruginosa,* and *K. pneumoniae*) and gram‐positive (*S. aureus* and *L. monocytogenes*) bacterial cultures were performed according to manufacturer instructions. The concentration of the extracted genomic DNA of each bacterial strain was determined using a micro‐volume UV–vis spectrophotometer (One Drop Touch Pro, Biometrics Technologies, USA). These extracted DNA samples were serially diluted in molecular grade water and used as a template for LAMP reaction.

##### LAMP Amplification of the Bacterial DNA

LAMP reactions (T100 thermal cycler, Bio‐Rad, USA) were performed with isolated genomic DNA using LavaLAMP DNA master mix (30 066‐1, Lucigen). Six primers (Table S1, Supporting Information) targeted *uidA* gene of *E. coli* was chosen from the literature.^[^ ^43^ ^]^ Primers were synthesized by Pro‐Tech chemicals, Taiwan. All samples were made in triplicate for testing. A 25 μL single LAMP reaction system was set up as mentioned in Table S2, Supporting Information. All LAMP reactions were performed at 70 °C. Amplification was confirmed by 1.5% agarose gel electrophoresis using a clear vision DNA stain (PT‐D1001, Pro‐Tech chemicals, Taiwan).

##### Principle of Rotational Diffusometry and the Relationship Between Viscosity and Nucleic Acid Amplification

Brownian motion is a natural phenomenon that can be defined as the random movement of a microscopic particle in suspension which is classified into translational and rotational. According to the Stokes–Einstein–Debye relation mentioned in Equation (1),^[^ ^35^ ^]^ the rotational diffusivity can be expressed as

(1) $$D_{r}=\frac{k_{B}T}{\pi \mu d_{p}^{3}}$$

where *T* is the temperature in kelvin, *μ* is fluid viscosity, *k*_B_ is Boltzmann constant, and the *d*_p_ is bead diameter. As described in Equation (1), the rotational diffusivity is inversely proportional to the fluid viscosity with constant temperature and particle diameter. Spherical particles are ideal for rotational diffusion compared to any other shape as it provides a higher rotational motion. On top of that, smaller sized particles (≤0.1 μm) deliver a better signal as the sedimentation due to gravity is much slower than the large particles. The limitations of this method are: 1) The uncertainty in the particles size contributes some deviation in the measurements; 2) Particle aggregation drops down the blinking intensity, resulting in a higher signal to noise ratio; 3) During the image capturing, particles drop out of the field of correlation rapidly, making the overall measurement process more difficult. These challenges can be overcome through proper dispersion of particles in the medium with appropriate sonication. Additionally, the particle size has to be small enough (≤0.1 μm) to avoid the rapid drop of particles from the field of correlation.

In this study, the rotational Brownian motion is investigated by using 1 μm sized half‐fluorescent and half‐gold coated functionalized Janus particles, which generate the blinking signal. LAMP was used for nucleic acid amplification and the effect of these LAMP‐amplified products in sample viscosity change was characterized using rotational diffusometry. In presence of appropriate LAMP components and the template DNA, an exponential nucleic acid amplification takes place, causing a significant increase in sample viscosity.^[^ ^36^, ^37^, ^38^ ^]^ Therefore, the diffusivity coefficient decreases and gives rise to a slow blinking signal by the functionalized Janus microbeads.^[^ ^39^ ^]^ However, without target DNA, the sample does not go under LAMP amplification, maintaining the sample viscosity constant. As a result, the diffusivity coefficient as well as the blinking signal of the particles remain stable.

A cross‐correlation algorithm was used to quantify the blinking signals generated by the Janus particles. The cross‐correlation algorithm can be defined as the calculation of particle's blinking frequency in a given pair of images (*I*_1_ and *I*_2_) at time 0 and 0 + Δ*t* by statistical methods, where each image is divided into four square‐gridded regions called interrogation windows to obtain a faster calculation. Calculations of the correlation intensity were performed by comparing the series of images of Janus microbeads with an increased time interval from 0 to 30 s, where the first image was fixed at 0 s but the second image was shifted by 0 + Δ*t* s. The cross‐correlation intensity was identical to the cross‐correlation function's peak value. With the increase of time interval, the peak value of the correlation intensity appeared to decline due to the loss of pairs. A slow decrease in the correlation intensity peak was observed in case of slow diffusion, on the other hand, correlation intensity rapidly drops in the event of strong diffusion. The cross‐correlation intensity is determined from the following equation

(2) $$R(s)=\int I_{1}(X)I_{2}(X+s)dX$$

where *X* is the coordinates in the image domain, and *s* is the coordinates in the correlation domain. The peak of the function, *R*(*s*), denotes the correlation intensity. The intensity diagram was then normalized from the following exponential regression

(3) $$A\text{exp}(-\frac{t}{\emptyset })+B$$

where ∅ is the characteristic correlation time of the intensity diagram, *A* and *B* are constants determined by fitting the exponential curve with data, and *t* is the elapsed time. The correlation time in this equation act for the exponential decay of the initially oriented population of particle with respect to time and it can be expressed as

(4) $$\emptyset =\frac{\mu V}{k_{B}T}$$

where *V* is the equivalent volume of the particles. Therefore, the correlation time is proportional to the fluid viscosity as the equivalent volume of microbead is fixed at a constant temperature.

##### Experimental Setup

At first, 1 μm sized Janus particles were thoroughly mixed with the LAMP‐amplified sample. Uniform dispersion of particles was ensured by 20 s of sonication. 2 μL of the mixture was pipetted on a glass slide. To create a sandwiched suspension, a glass coverslip was placed on top of the sample droplet with a spacer of 110 μm and positioned under an IX71 fluorescent microscope (Olympus, Tokyo, Japan) with a 10x objective. CCD camera (Firefly MV FMVU‐13S2C, Point Grey, Richmond, BC, Canada) was used to capture a series of images of the microbeads in the suspension at a frame rate of 10 Hz. Blinking signals generated from the Janus microbeads were measured and the cross‐correlation analysis was performed using MATLAB code. Six measurements of 300 images each were performed for every sample and the characteristics correlation times were plotted.

##### Real‐Time PCR Analysis

Real‐time PCR reactions (Step one plus system, Applied Biosystems) were performed with isolated genomic DNA using Fast‐start universal SYBR green master (rox) (4 913 914 001, Roche, Germany). Primers with an amplicon size of 147 bp (Table S3, Supporting Information) for the targeted *uidA* gene of *E. coli* were chosen from the literature.^[^ ^44^ ^]^ Primers were synthesized by Pro‐Tech chemicals, Taiwan. All samples were made in triplicate for testing. A 20 μL single qPCR reaction system was set up (Table S4, Supporting Information) and performed with an initial denaturing step at 95 °C for 10 min, followed by 40 cycles of denaturation at 95 °C for 15 s, annealing and extension at 60 °C for 60 s followed by a thermal denaturation protocol. Real‐time PCR results were analyzed and the cycle threshold (C_T_) values were determined with Sequence Detection system software (Applied Biosystems).

##### Food and Water Sample Analysis

Milk and juice samples were purchased from the supermarket and used as test samples. These samples were initially centrifuged twice at 8000 rpm to remove supernatant, which includes fat (milk) and other solid particles, respectively. Water and the food samples were artificially spiked with *E. coli* and cultured for several hours. 2 mL aliquot from water, milk, and juice were centrifuged at 5000 rpm for 3 min and the bacterial cells were extracted and redissolved in molecular biology grade water and used for LAMP reaction. The LAMP reactions were performed as mentioned in the previous section.

## Conflict of Interest

The authors declare no conflict of interest.

## Supporting information

Supplementary Material

## Data Availability

The data that support the findings of this study are available in the supplementary material of this article.
