# Supplementary material for: Ultrafast and Sensitive Screening of Pathogens by Functionalized Janus Microbeads‐Enabled Rotational Diffusometry in Combination with Isothermal Amplification
Source: Small Sci. 2022 Mar 3;2(5):2200010. doi: 10.1002/smsc.202200010 (PMC11935808; doi:10.1002/smsc.202200010)
Supplement: Supplementary file 1 — Supplementary Material [file SMSC-2-2200010-s001.zip › Revised_Supporting_information_(clean).pdf]

Supporting information

Ultra-fast and Sensitive Screening of Pathogens by Functionalized Janus Microbeads Enabled Rotational Diffusometry in Combination with Isothermal Amplification

*Dhrubajyoti Das [1], Hui-Chen Hsieh [2,3], Chang-Shi Chen [2,3], Wei-Long Chen [1], Han-Sheng Chuang [1,4]**

[1]*Department of Biomedical Engineering, National Cheng Kung University, Tainan 701, Taiwan*

[2]*Department of Biochemistry and Molecular Biology, National Cheng Kung University, Tainan 701, Taiwan*

[3]*Institute of Basic Medical Sciences, College of Medicine, National Cheng Kung University, Tainan 701, Taiwan*

[4]*Medical Device Innovation Centre, National Cheng Kung University, Tainan 701, Taiwan*

* *Correspondence:* oswaldchuang@mail.ncku.edu.tw

Contents:

1. **Figure S1:** Gel Electrophoresis image of *E. coli* LAMP amplification in different temperature. (a) 70 ºC (b) 65 ºC…………………………………………………….....S2
2. **Figure S2:** Gel Electrophoresis image of LAMP amplification of extracted *E. coli* whole cells in (a) water (b) milk and (c) juice. The LAMP reaction is shown for three different time - 10, 20 and 30 min to confirm the amplification…………………………………………………………………….....S3
3. **Figure S3:** Standard curve and the linear regression value for the series of dilution of DNA concentration (10^n fg/µL)…………………………………………………....S3
4. **Figure S4:** Correlation functions and their peak values at (a) 0 s and (b) 20 s. (c) Cross-correlation intensity decreases with time. The black and red curves refer to the control and (+) sample, respectively………………………………………………………..S4
5. **Figure S5-7:** Intensity distribution plot from DLS analysis of 1 µm polystyrene particles..………………………………………………………………………S7-9
6. **Figure S8:** (a) The blinking frequency of a single particle of the simulation. (b) Cross-correlation intensity plot of the simulation...S9

7. **Figure S9:** Images sequence of flow of control and (+) sample in capillary tubes........S10
8. **Table S1:** LAMP primer sequences for the target gene………………………………S4
9. **Table S2:** Single LAMP reaction System…………………………………………….S5
10. **Table S3:** PCR primer sequences for the target genes………………………………..S5
11. **Table S4:** Single qPCR System……………………………….………………...S6
12. **Table S5:** DLS analysis for 1 μm polystyrene particles……………………………...S6
13. **Video S1:** Simulation video of 1 μm Janus particles creating blinking signal at magnification of 40x………………………………………………………………S10
14. **Video S2:** Flow of control and (+) sample in capillary tubes…………………………S10
15. Visual analysis of flowrate of LAMP amplified sample………………………………S10

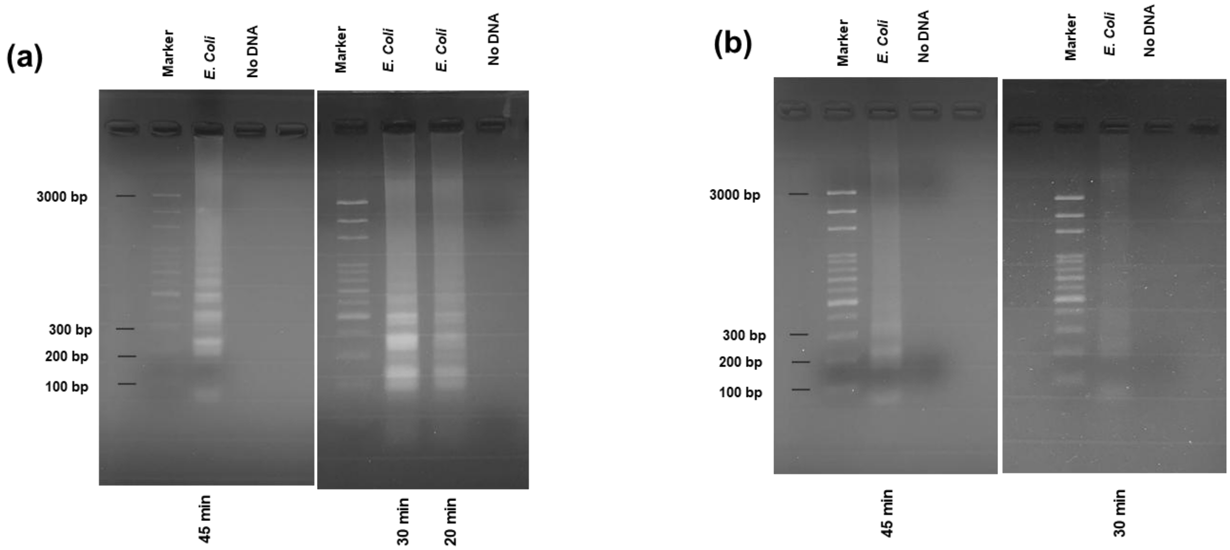

Figure S1: Gel Electrophoresis image of *E. coli* LAMP amplification in different temperature. (a) 70 ºC (b) 65 ºC

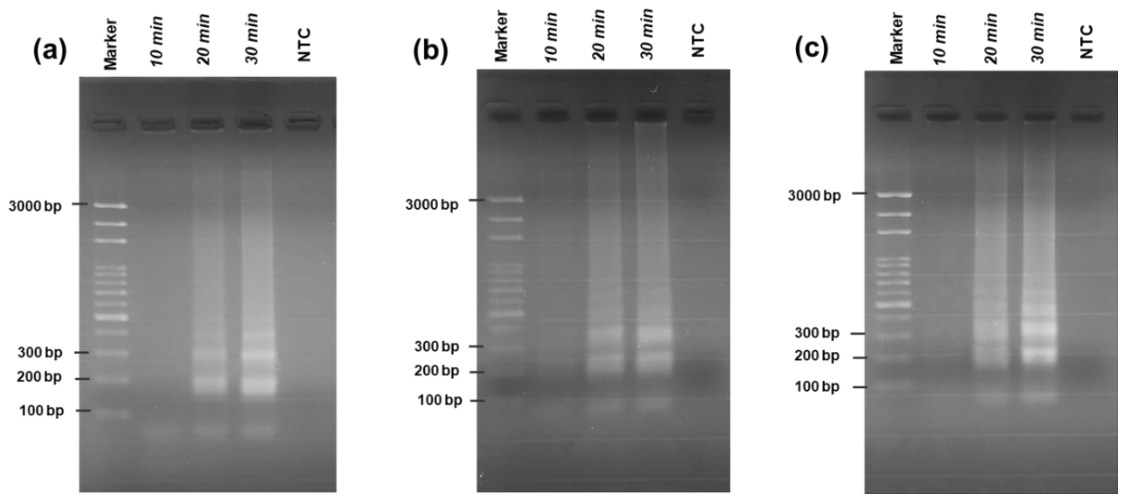

Figure S2: Gel Electrophoresis image of LAMP amplification of extracted *E. coli* whole cells in (a) water (b) milk and (c) juice. The LAMP reaction is shown for three different time - 10, 20 and 30 min to confirm the amplification.

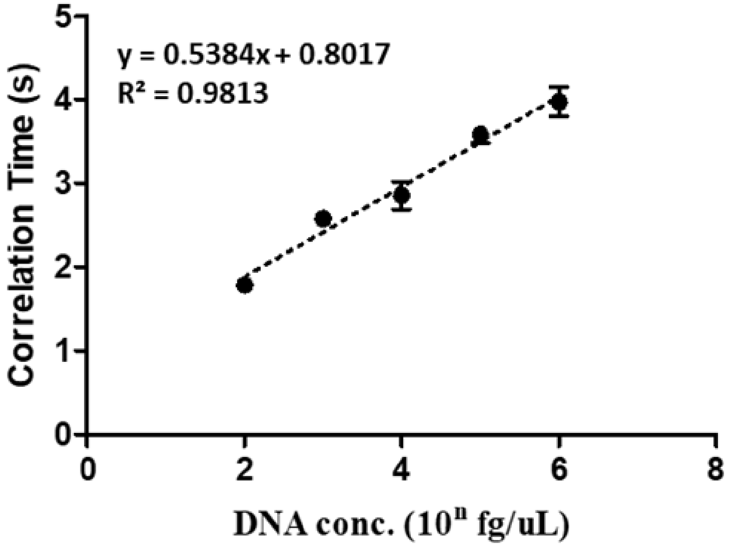

Figure S3: Standard curve and the linear regression value for the series of dilution of DNA concentration (10^n fg/µL)

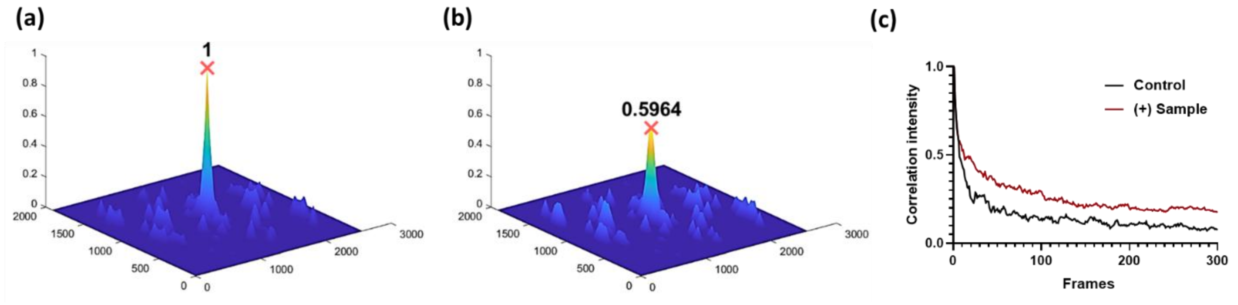

Figure S4. Correlation functions and their peak values at (a) 0 s and (b) 20 s. (c) Exponential decay of cross-correlation intensity. The black and red curves refer to the control and (+) sample, respectively.

Table S1. LAMP primer sequences for the target gene

Gene	Primer Sequence
uidA	FIP- 5′-TAACGCGCTTTCCCACCAACGGCCTGTGGGCATTCAGTC-3′<br>BIP- 5′-TAACGATCAGTTCGCCGATGCACTGCCCAACCTTTCGGTAT-3′<br>F3- 5′-CKGTAGAAACCCCAACCCG-3′<br>B3- 5′-AWACGCAGCACGATACGC-3′<br>LF- 5′-TCCACAGTTTTCGCGATCCA-3′<br>LB- 5′-ACGTCTGGTATCAGCGCGAAGT-3′

Table S2. Single LAMP reaction System

Reagents	Concentration
LAVALAMP DNA Master mix	12.5 µL
F3 Primer	0.2 µM
B3 Primer	0.2 µM
FIP Primer	1.6 µM
BIP Primer	1.6 µM
LB Primer	1.6 µM
LF Primer	1.6 µM
Sample	1 µL
ddH_2O added to reach	25 µL

Table S3. PCR primer sequences for the target genes

Gene	Primer Sequence	Amplicon length
uidA	Forward primer: 5'-AAAAC GGCAA GAAAA AGCAG-3'<br>Reverse primer: 5'-ACGCGTGGTTACAGTCTTGCG-3'	147 bp

Table S4. Single qPCR System

Reagents	Concentration
Sample	1 μL
Forward (10 μM)	0.4 μL
Reverse (10 μM)	0.4 μL
SYBR Green Mix	10 μL
ddH_2O added to reach	20 μL

Table S5: DLS analysis for 1 µm polystyrene particles

No	Mean diameter (nm)	Polydispersity index
1	1030.5	-0.049
2	996.9	-0.039
3	1026.8	-0.046

BECKMAN COULTER

Delsa™ Nano
Common

Intensity Distribution S/N :

User	: Common	Group	:	Repetition	: 1/3
Date	: 5/14/2019	File Name	: 20190514-poly-1_20190514_105557		
Time	: 10:55:57	Sample Information	:		
SOP Name	: 20160922 Elisa			Security	: No Security

Version 2.31 / 2.03

Intensity Distribution

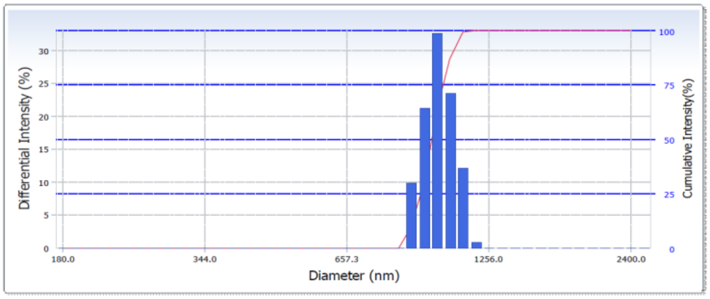

Distribution Results (Nnls)

Peak	Diameter (nm)	Std. Dev.
1	1,000.1	69.5
2	0.0	0.0
3	0.0	0.0
4	0.0	0.0
5	0.0	0.0
Average	1,000.1	69.5
Residual :	8.657e-003	(O.K)

Cumulants Results

Diameter	(d)	: 1030.5	(nm)
Polydispersity Index	(P.I.)	: -0.049	
Diffusion Const.	(D)	: 4.159e-009	(cm²/sec)
		:	

Measurement Condition

Temperature	: 20.0	(°C)
Diluent Name	: WATER	
Refractive Index	: 1.3334	
Viscosity	: 1.0020	(cP)
Scattering Intensity	: 10739	(cps)

Figure S5: Intensity distribution plot from DLS analysis of 1 µm polystyrene particles.

BECKMAN COULTER

Delsa™ Nano
Common

Intensity Distribution S/N :

User	: Common	Group	:	Repetition : 2/3	
Date	: 5/14/2019	File Name	: 20190514-poly-1_20190514_105557		
Time	: 10:55:57	Sample Information	:		
SOP Name	: 20160922 Elisa			Security	: No Security

Version 2.31 / 2.03

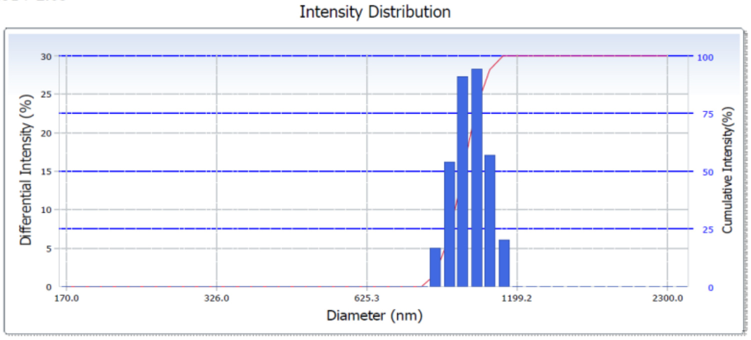

Distribution Results (Nnls)

Peak	Diameter (nm)	Std. Dev.
1	980.0	72.8
2	0.0	0.0
3	0.0	0.0
4	0.0	0.0
5	0.0	0.0
Average	980.0	72.8
Residual :	7.458e-003	(O.K)

Cumulants Results

Diameter	(d)	: 996.9	(nm)
Polydispersity Index	(P.I.)	: -0.039	
Diffusion Const.	(D)	: 4.299e-009	(cm²/sec)
		:	

Measurement Condition

Temperature	: 20.0	(°C)
Diluent Name	: WATER	
Refractive Index	: 1.3334	
Viscosity	: 1.0020	(cP)
Scattering Intensity	: 12703	(cps)

Figure S6: Intensity distribution plot DLS analysis of 1 µm polystyrene particles.

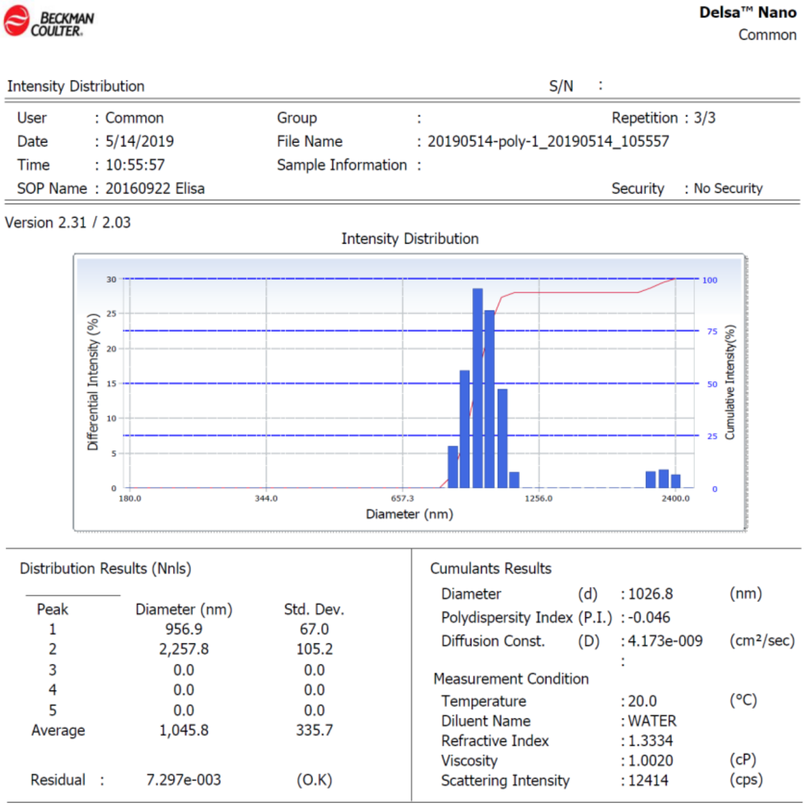
BECKMAN COULTER

Delsa™ Nano
Common

Intensity Distribution S/N :

User	: Common	Group	:	Repetition	: 3/3
Date	: 5/14/2019	File Name	: 20190514-poly-1_20190514_105557		
Time	: 10:55:57	Sample Information	:		
SOP Name	: 20160922 Elisa			Security	: No Security

Version 2.31 / 2.03

Distribution Results (Nnls)

Peak	Diameter (nm)	Std. Dev.
1	956.9	67.0
2	2,257.8	105.2
3	0.0	0.0
4	0.0	0.0
5	0.0	0.0
Average	1,045.8	335.7

Residual : 7.297e-003 (O.K)

Cumulants Results

Diameter	(d)	: 1026.8	(nm)
Polydispersity Index	(P.I.)	: -0.046	
Diffusion Const.	(D)	: 4.173e-009	(cm²/sec)
		:	

Measurement Condition

Temperature	: 20.0	(°C)
Diluent Name	: WATER	
Refractive Index	: 1.3334	
Viscosity	: 1.0020	(cP)
Scattering Intensity	: 12414	(cps)

Figure S7: Intensity distribution plot DLS analysis of 1 µm polystyrene particles.

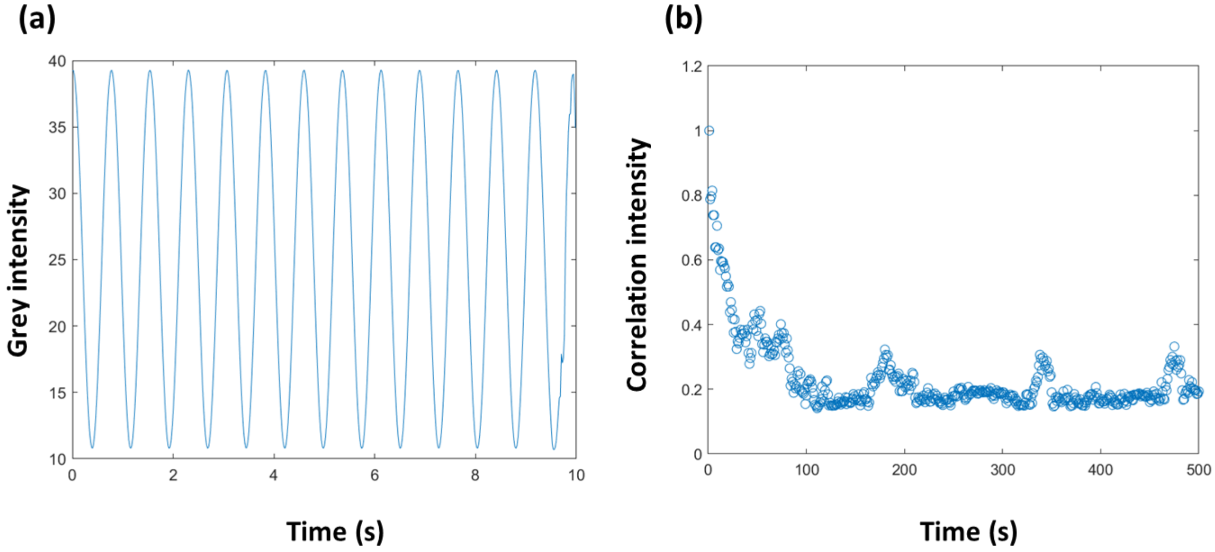

Figure S8: (a) The blinking frequency of a single particle of the simulation. (b) Cross-correlation intensity plot of the simulation.

1. Visual analysis of flowrate of LAMP amplified sample:

5 µL of control (sample undergone LAMP amplification without any *E. coli* genomic DNA) and the 5 µL of (+) sample (sample undergone LAMP amplification with 10 ng/µL of *E. coli* genomic DNA for 45 min) solution were loaded in two different capillary tube fixed with a metal clamp. Then both the capillary tube together kept at an angle of 45º-65º. The flow of the liquid was recorded via cellphone camera (Figure S8). The images of the sample flowing through the capillary tube was recoded with 2 s time interval. From the images it was clearly visible that the sample contains the LAMP amplified DNA travel slower than the control. This indicates that the (+) sample viscosity increases due to the presence of amplified DNA resulting a slow flowrate of the sample.

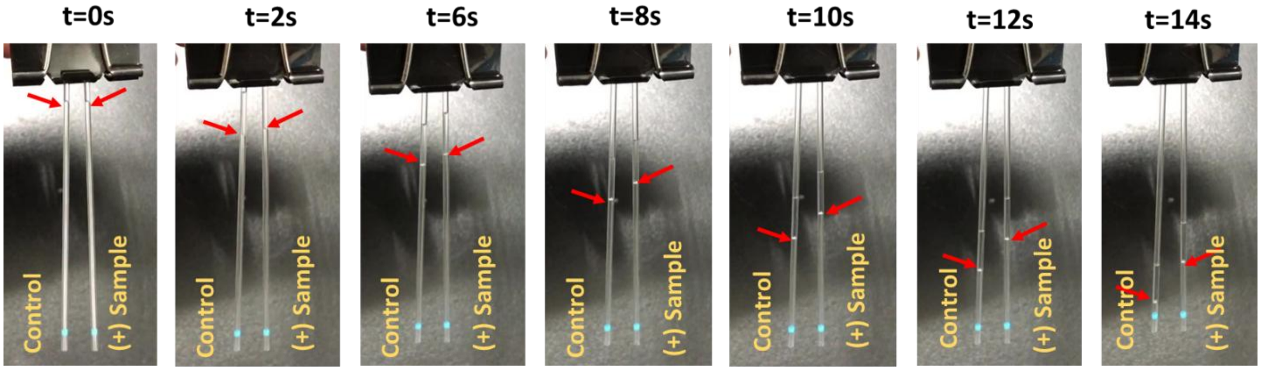

Figure S9: Images sequence of flow of control and (+) sample in capillary tubes.

Supporting video:

Video S1: Simulation video of 1 µm Janus particles creating blinking signal at magnification of 40x.

Video S2: Comparison of the flow of control and (+) sample in capillary tubes
